# Genetic variation in Japanese Holstein cattle for EBL development

**DOI:** 10.1186/s12917-020-02625-8

**Published:** 2020-10-28

**Authors:** Yasuko Inagaki, Tomoko Kobayashi, Yoshihito Suda, Kazuya Kusama, Kazuhiko Imakawa

**Affiliations:** 1 Laboratory of Animal Health, Department of Animal Science, Faculty of Agriculture, Tokyo University of Agriculture, 243-0034 Atsugi, Kanagawa Japan; 2 Department of Food, Agriculture and Environment, Miyagi University, 982-0215 Sendai, Miyagi Japan; 3 Department of Endocrine Pharmacology, Tokyo University of Pharmacy and Life Sciences, 192-0392 Tokyo, Japan; 4 Laboratory of Molecular Reproduction, Research Institute of Agriculture, Tokai University, 862-8652 Kumamoto, Japan

**Keywords:** Bovine leukemia virus, Enzootic bovine leukosis, Holstein cattle, heritability

## Abstract

**Background:**

Infection with bovine leukemia virus (BLV), the causative agent for enzootic bovine leukosis (EBL), is increasing in dairy farms of Japan. The tendency of tumor development following BLV infection in certain cow families and bull lines has previously been described. We therefore hypothesized the existence of a genetic component which differentiates cattle susceptibility to the disease.

**Results:**

We analyzed routinely collected large-scale data including postmortem inspection data, which were combined with pedigree information and epidemiological data of BLV infection. A total of 6,022 postmortem inspection records of Holstein cattle, raised on 226 farms served by a regional abattoir over 10 years from 2004 to 2015, were analyzed for associations between sire information and EBL development. We then identified statistically the relative susceptibility to EBL development for the progeny of specific sires and paternal grandsires (PGSs). The heritability of EBL development was calculated as 0.19. Similarly, proviral loads (PVLs) of progeny from identified sires and PGSs were analyzed, but no significant differences were found.

**Conclusions:**

These observations suggest that because EBL development in our Holstein population is, at least in part, influenced by genetic factors independent of PVL levels, genetic improvement for lower incidence of EBL development in cattle notwithstanding BLV infection is possible.

## Background

Among the many environmental and managemental factors affecting dairy cattle production, enzootic bovine leukosis (EBL), caused by the bovine leukemia virus (BLV) [1], an oncogenic retrovirus, undoubtedly has a negative impact on dairy productivity. It is now believed that 79.1% of dairy farms and 34.7% of dairy cattle in Japan are infected with BLV [2, 3]. BLV is transmitted mainly by transferring infected blood through contaminated needles, rectal palpation sleeves, dehorners or bloodsucking insects. Because infected cattle are lifelong carriers and remain a reservoir of infection for other cattle, reducing herd BLV prevalence through segregation or gradual culling of BLV-positive animals is a lengthy process. The high prevalence of BLV may affect cattle farms by decreasing milk yield and necessitating carcass condemnation at slaughterhouses [4]. The number of reported cases of Bovine Leukosis (BL), which includes EBL and Sporadic Bovine Leukosis (SBL), has been increasing every year since BL was designated as a notifiable disease in Japan in 1998.

The possibility of genetic contributions to susceptibility to BL has been reported in previous studies [5–7]. Although about 1 to 5% of BLV infected cattle develop lymphosarcoma at ages 4 to 5, more than 90% of them remain asymptomatic throughout their productive life [7]. A recent study indicated that although increasing age and high proviral load (PVL) increase risks of EBL development, not all aged cattle with higher PVL develop neoplastic tumors. Rather, only 14% of the high risk group with age older than 4.5 years and PVL higher than 52,000 copies/10^5^ cells were diagnosed with EBL [8]. Genetic components that promote tumor development from both paternal and maternal pedigrees have been described in several previous reports published in the middle of the 20th century, before the identification of BLV as the causative agent of EBL. These previous reports have shown evidence of a family of cattle highly susceptible to lymphoma development that all descended from one bull in East Prussia [9], a significant difference in predisposition between two bulls in the same herd [10], and a significant positive correlation between daughters and dams with regard to the development of leukotic tumors [11]. These reports indicate that the susceptibility to the development of neoplastic tumors, now known to be EBL, could be heritable in cattle populations. However, as these data are based on only a few herds with a small data set, it is difficult to statistically evaluate the genetic variability associated with EBL development among pedigrees. Moreover, such a small data set makes it difficult to eliminate the influence of environmental factors on EBL development.

Since the year 2003, the Japanese dairy industry has participated in the International Bull Evaluation Service (INTERBULL) program, and male stock information has been accumulated in the Domestic Animal Improvement (DAI) databank of Japan. In 2003, a national program under the Cattle Traceability Law was implemented to trace each animal from birth to slaughter. All cattle were mandatorily registered with an individual identification (ID) number in the database established at the National Livestock Breeding Center (Fukushima, Japan). Each cattle ID entry retains extensive information, including dam ID, breed, sex and all translocation history from birth to slaughter, and is linked with the DAI databank. In 1998, bovine leukosis was added to the list of reportable animal diseases in the Domestic Animal Infectious Diseases Control Law. Subsequently, cattle diagnosed as EBL were subjected to compulsory destruction, and diagnostic information at the time of postmortem inspection was stored by the veterinary officers of Municipal Meat Inspection Stations.

The objective of this study was to use these data to assess the information supporting a genetic variation regarding EBL development as well as BLV infection in Holstein cattle, and to gain insight into the mode of inheritance. These objectives were examined through a case-control study of the Holstein cattle registered in the DAI databank for the period of 2004 to 2015.

## Results

### Study 1**-**Analysis of postmortem inspection data

**Fig. 1:**
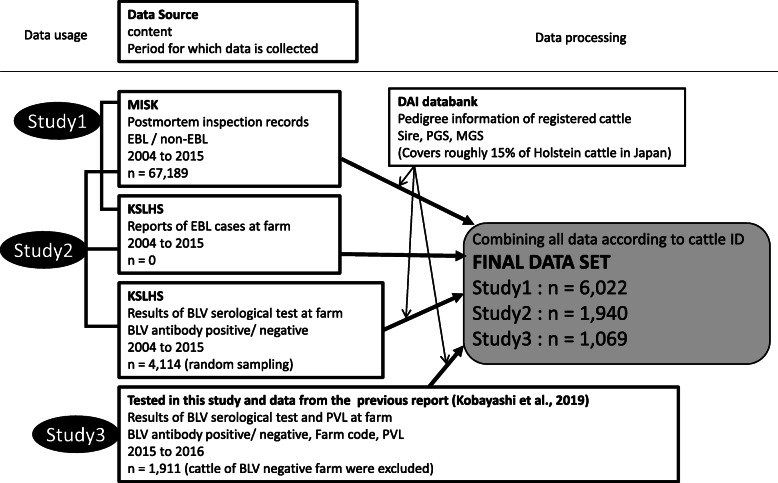
Schematic overview of the analysis on cattle data The data from four data sources were combined according to cattle ID, and the cattle data of which ID was registered to DAI data bank was further extracted. KSLHS: Kanagawa Shonan Livestock Hygiene Station, MISK: Meat Inspection Station, Kanagawa Prefectural Government, DAI: Domestic Animal Improvement, PGS: Paternal grandsire, MGS: Maternal grand sire

Postmortem inspection records of 6,022 Holstein cattle raised on 226 farms were analyzed for associations between pedigree and EBL development (Fig. 1). Among this study population, 69 cattle were diagnosed as EBL at the local abattoir, while the remaining cattle exhibited no symptoms of development of lymphoid tumors (Table 1). In this study area, none of the cattle were reported as EBL during the study period and thus all EBL diagnosis was made at the abattoir. From cattle IDs, pedigree information including sire, paternal grand sire (PGS) and maternal grand sire (MGS) of each animal were analyzed. The 6,022 Holstein cattle analyzed in Study1 were sired by 1,070 bulls (defined in this study as Sire). These Sires were bred by 288 bulls (defined as PGS). Regarding maternal lineage, although the lineage information was not available for dams of 51 cattle and grandsires for 291 cattle among 6,022 cattle, 5,971 cattle were born from 4,921 dams. These dams were sired by more than 958 sires (defined as MGS). For the EBL case population, 52 sires, 39 PGSs and more than 53 MGSs were identified. All sires with at least 50 daughters or granddaughters were included in the case/control file, from which tables were built and Fisher’s exact test was performed to compare cell contents and compute the odds ratio (OR) for each sire (Table 1). In this analysis, each sire/grandsire was compared against all other sires and grandsires. ORs are presented as 95% confidence intervals. Among all sires of the study population, 13 sires had daughters numbering at least 50 heads. Sire9 and Sire13 present significantly high frequencies of EBL cases (OR = 3.99 (1.04–12.24), *p* = 0.046 and OR = 6.04 (1.95–16.97), *p* = 0.006). PGS1 and PGS3 present significantly low frequencies of EBL cases (*p* = 0.033 and *p* = 0.049), while PGS6 and PGS12 present significantly high frequencies of EBL cases (OR = 2.52 (1.05–5.95), *p* = 0.042 and OR = 3.07 (1.00–8.24), *p* = 0.049) in the granddaughter population. None of the MGS exhibited a significant difference in the development of EBL in their progenies (Table 1). There are no significant differences in longevity between cattle that developed lymphoma and cattle that did not develop lymphoma (5.64 years vs. 5.96 years, *p* = 0.154, Mann-Whitney U Test).

**Table 1:**
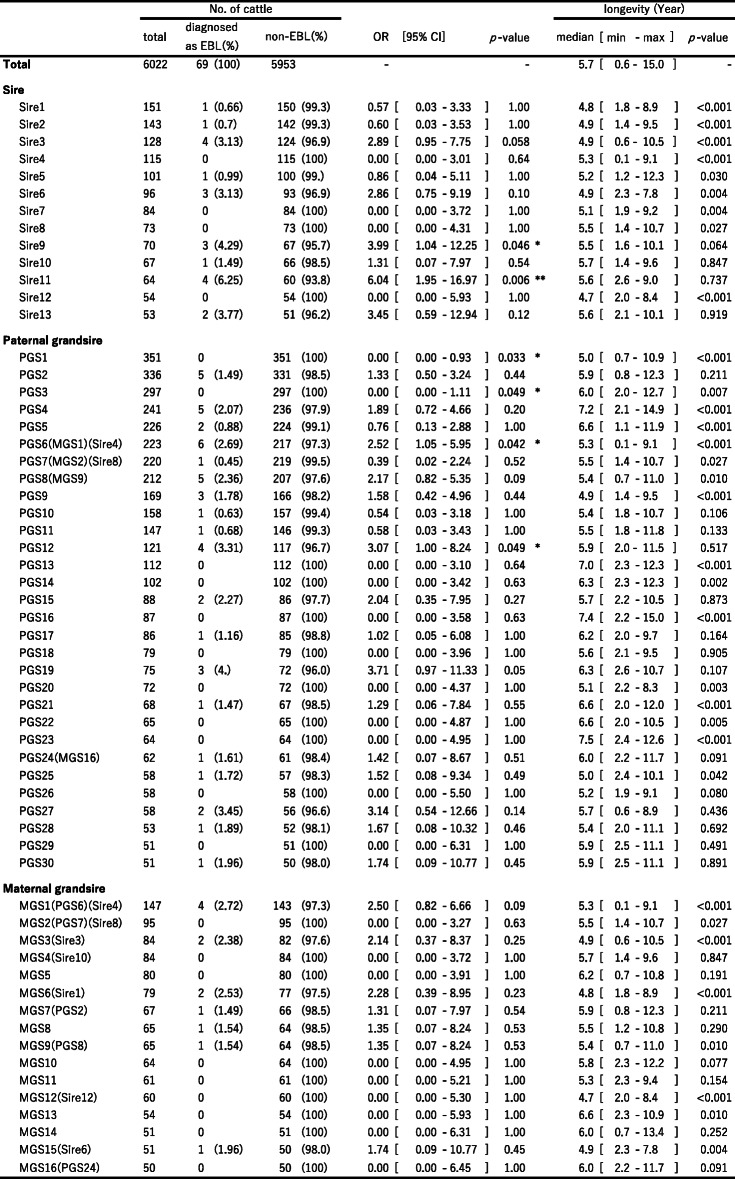
No. of daughters/granddaughters slaughtered and examined for EBL at postmortem inspection listed by sire/grandsire

*p<0.05 **p<0.01 *OR *odds ratio; *CI* confidence interval

### Study 2-Heritability estimate of BLV infection and EBL susceptibility

Based on the data of Study 1, we identified the sires and PGSs that exhibited significant differences in the development of EBL in their daughter and granddaughter populations. These data indicated that some lineages either more susceptible or less susceptible to EBL tumor development could exist. To estimate the heritability of BLV infection and EBL susceptibility among Holstein cows, the dataset from IDs was linked to the lineage information available at the DAI databank. In this analysis, the heritability was calculated by using the BLUP method; the diagnostic information regarding EBL and the presence or absence of BLV antibodies were treated as random effects, while age at analysis, the year of analysis, on-farm BLV infection and EBL development were treated as fixed effects. The heritability of EBL development was 0.19, where the additive genetic variance (${\sigma }_{g}^{2})$ was 0.0508 and the residual variance (${\sigma }_{r}^{2})$ was 0.222.

### Study 3- PVL of progenies of detected sires and grandsires at farm level

Previously, it was found that cattle exhibiting disease-related tumors following BLV infection had higher PVLs [8, 12]. The question then arises whether the difference in EBL frequencies among pedigrees is associated with PVL at the individual animal level. Therefore, we analyzed the data of PVL levels of progenies of identified sires and PGSs. The PVL of these cattle ranged from zero to 374,025 copies/10^5^ cells, the median was 42,072 copies/10^5^ cells (Table 2). There was no significant difference in PVL levels between the progenies of the identified sires and PGSs and the progenies of other sires and grandsires.

**Table 2:** Seroprevalence and PVL per sires with significant odds ratio

	Sero-prevalence				PVL (copies/10^5^ cells)		
	total	negative	positive	%	median	min-max	*p-value*
total	1069	461	608	56.9	42072	[0 -374025]	-
Sire9	2	1	1	50.0	66464	[66464 - 66464	0.459
Sire11	8	0	8	100.0	4134	[0-75336]	0.077
other Sires	1059	460	599	56.6	48140	[0-374025]	-
PGS1	33	11	22	66.7	47672	[0-137995]	0.808
PGS3	8	2	6	75.0	44952	[18576-76227]	0.757
PGS6	36	10	26	72.2	52988	[0-117272]	0.701
PGS12	5	2	3	60.0	51901	[0-98155]	1
other PGSs	987	436	551	55.8	47943	[0 -374025]	-

## Discussion

Most BLV-infected animals remain asymptomatic, although a small fraction of infected animals exhibit tumor development during the course of disease progression. The tendency for tumor development after BLV infection in certain cow families and bull lines has previously been described [9–11]. However, there has been no statistical evidence to support the existence of genetic components that influence the degree of susceptibility to the disease. In this study, we analyzed routinely collected large-scale data including postmortem inspection data, which were combined with EBL diagnostic data and pedigree information. We then identified statistically the sires and PGSs that were more susceptible or less susceptible to the development of EBL (Table 1). These findings were made possible because the information related to BLV epidemiological data at farms and EBL diagnostic data at the local abattoir were all linked through each cattle ID. Based on this information, it was possible to estimate the heritability of susceptibility to the BLV infection and EBL development as 0.08 and 0.19, respectively. The former estimate agrees with the heritability calculated from BLV prevalence in milk [5]. These observations suggest that development of EBL in our Holstein population is, at least in part, influenced by genetic factors.

Previous studies have shown that the specific alleles of the major histocompatibility complex, bovine leukocyte antigen (BoLA) genes, are significantly associated with PVL in BLV-infected animals. BoLA-DRB3*009:02, BoLA-DRB3*002:01 and DRB3*014:01:01 are reported as the alleles associated with low PVL in several studies [13–15]. In the population examined in Study 3, we analyzed the BoLA DRB3 haplotype of granddaughters of identified PGSs. However, paternal granddaughters with these particular alleles were found in only 23.3% (BoLA-DRB3*009:02: 6.7%, BoLA-DRB3*002:01: 0% and DRB3*014:01:01: 20%) of those examined (data not shown). In addition, as 99% of Holstein cows were asymptomatic, these results indicate that genetic factors that affect disease development may be not only BoLA but also others yet to be identified.

A limitation of our study is that in the analysis of Study 1, we assessed the frequency of EBL development at the time of slaughtering. It is possible that the difference of the frequency of EBL development could be biased by cattle that were slaughtered before the age of tumor development. In the population of Study 1, the median longevities of the progenies of PGS1 which display no EBL development was slightly shorter than that of other PGSs (5.0 years vs. 5.7 years, p < 0.001, Mann-Whitney U test). This may have biased the results and should be noted when interpreting the data. However, the differences in longevity of the progenies of PGS1 are less than a year, and do not fully explain the absence of EBL cattle because the age of occurrence of EBL ranges from 1.6 years to 9.8 years in this study.

In our study, Holstein cattle were from a small region in Japan, but ID numbers and male stock information accumulated in the DAI data bank enabled us to trace definitive information on individual animals, sire information and EBL development. It should be noted that BLV seroprevalence rates in individual cows and farms were similar, if not the same, to those reported for the entire population of Holstein cattle in Japan [3]. The seropositive rates were also similar to those of the US [16, 17] and Canada [4] due at least in part to Japan’s participation in INTERBULL since 2003. These data indicate that Holstein sire usage is similar in these countries, exhibiting genetic homology and homogeneity. It was reported that genetic correlation is increasing in dairy countries, resulting from the use of genetically superior sires [18]. VanRaden et al. [19] also reported that genetic makeups of Holstein sires in major dairy countries including Japan are similar to those of the US. Identification of the sires and PGSs with reduced frequency of EBL development in their progenies suggests that sires potentially identified through the use of methods tested in this study could be applied to reduce economic losses related to EBL in major dairy countries.

It is still an open question as to whether focusing on prevention of EBL development through genetic selection rather than reduction in BLV infection or control of PVL levels once infected is most beneficial to dairy production and/or population. In this study, all EBL cases were found at a local abattoir and therefore most of the dairy cattle infected with BLV had survived through their productive lives without exhibiting clinical symptoms or fatal lymphoma. Our observations indicate that sire selection from the grandsires less susceptible to developing EBL in their progenies could serve as one method in reducing EBL-incidence in dairy producing countries.

## Conclusions

In this study, we demonstrated that among the many sires used in Japan, there exist sires and PGSs exhibiting increased or decreased frequency of tumor development in their progenies, although PVL did not differ among the progenies of sires and PGSs examined. The observed level of heritability indicates that genetic contributions are of importance in EBL development in Holstein cattle. Although the sources of genetic variability or patterns of inheritance have not been elucidated, the use of PGSs that are less susceptible to tumor development could reduce the frequency of EBL in the cattle population. It is possible that more such PGSs could be identified if analyses similar to this study are carried out elsewhere in the world.

## Methods

The investigation was divided into three components, as follows:

### Study 1 **-** Analysis of postmortem inspection data

#### Study area and data collection

In this study, we used the data from a local abattoir within jurisdiction of the Meat Inspection Station, Kanagawa Prefectural Government (MISK), as collected from 2004 to 2015. This abattoir receives 4,000 to 5,000 cattle annually for slaughtering, mainly from Kanagawa prefecture and nearby Shizuoka prefecture, both of which are located in the central part of Japan near the capital of Tokyo. The diagnostic data of the postmortem inspection of all slaughtered cattle were recorded with each cattle ID by the veterinary officers. A total of 67,189 inspection records were included in the data set before editing. Data were further edited to include only the records of Holstein cattle received at the local abattoir from farms within the jurisdiction of the Kanagawa Shonan Livestock Hygiene Service Center (KSLHS), so that information of the cattle diagnosed with EBL in these farms was also available. Additionally, data were further edited to include only Holstein cattle registered to the DAI databank so that pedigree information could be examined. A total of 6,022 postmortem records were included in the final data set (Fig. 1). These data constituted the case group (all cattle diagnosed with EBL) and the control group (all remaining cattle), and tabulated by pedigree information (sire, PGS, and MGS).

#### EBL diagnosis

For all animals slaughtered at the local abattoir, when neoplastic lesions are found at inspection, the carcass is separately stored and further laboratory examinations are performed: lymphoma or lymphosarcoma is diagnosed by histologic examination, followed by additional immunohistochemical analysis to determine T-cell or B-cell origin. Subsequently, antibodies against BLV in serum are detected with a passive hemagglutination test (Nisseiken Co., Ltd., Ome, Japan).

### Study 2**-**Heritability estimate of BLV infection and EBL development

#### Study area

During the course of data collection in Study 1, a serological survey of 4,114 randomly selected cattle was performed at the KSLHS. The results of serological testing were cross-referenced with the final data set of Study 1 to extract cattle ID found in both data sets. These IDs were tabulated with results of the BLV serological test and diagnostic information regarding development of EBL, from which their pedigrees were further evaluated (Fig. 1).

#### Heritability estimate

A total of 1,940 cattle confirmed through ID to be present in all three relevant databases, namely the serological test, postmortem inspection data and DAI data banks, were selected for this study. Based on this information, the heritability for EBL development was calculated using a threshold model, presented in matrix notation as y = Xβ + Zα + e, where y is a vector of observations, β is a vector with the fixed effects of year of birth (17 levels), age of testing (13 levels) and 98 farms (3 prevalence levels), α is a vector of additive genetic effects (i.e., breeding values), e is a vector of residual effects, and X and Z are prevalence matrices that link individual susceptibility to the BLV infection and development of lymphoid tumors to their respective fixed and random effects.

Using the THRGIBBS1F90 software program [20], Gibbs sampling programs for categorical traits and Bayesian analysis were performed to estimate the variance components in the models. Single chains of 100,000 cycles were run with the first 50,000 cycles used as the burn-in period, followed by post-Gibbs analysis using the POSTGIBBSF90 software program [21]. These repeated analyses enabled the estimation of (co)variance, G-matrix and R-matrix, resulting in the calculation of the heritability for BLV infection and development of EBL. In this analysis, there was no significant difference between sire usage in the study area and that of Japan as a whole (data not shown).

### Study 3-PVL of progenies of sires on farm

#### Study area and sample collection

To analyze whether or not genetic variability is associated with susceptibility to BLV infection and/or PVL levels, the epidemiological data of daughters/granddaughters of identified sires/PGSs were obtained from the survey conducted within the same study area in 2015. Sampling and testing procedures were performed as previously described in detail [8]. In addition to the previous data from only the year 2015, blood samples were further collected using the same methods from a neighboring city in 2016. Serological tests were conducted and PVL levels of seropositive cattle were measured. The results of the epidemiological surveys for the two cities were analyzed together. A total of 2,179 cattle from 58 farms were included in the final data set. Among these data, cattle raised in farms without seropositive animals were excluded. Additionally, data were further edited to include only Holstein cattle registered to the DAI databank. A total of 1,069 cattle data from 49 farms were included in the final data set (Fig. 1). These records were tabulated by the results of serological test, PVL levels for seropositive animals, and distinct pedigrees.

#### Statistical analysis

Odds ratio (OR) was estimated by Fisher’s exact test. Longevity for each cow was calculated from the date of birth to the date of culling or date of death. Longevities and BLV PVLs (copy numbers/10^5^ cells) were used as a continuous variable. Continuous data was presented as median (range) values and compared using a Mann-Whitney U test. All statistical analyses were performed with SPSS, R version 3.2.1 (The R Foundation for Statistical Computing, Vienna, Austria) and EZR version 1.36 (Saitama Medical Center, Jichi Medical University, Saitama, Japan), which is a graphical user interface for R.

## Data Availability

The datasets used and analysed during the current study are available from the corresponding author on reasonable request and with permission of Kanagawa Prefectural Government.

## List of Abbreviations

EBL: Enzootic Bovine Leukosis
BLV: Bovine Leukemia Virus
BL: Bovine Leukosis
SBL: Sporadic Bovine Leukosis
PVL: Proviral load
DAI: Domestic Animal Improvement
ID: Individual identification
MISK: Meat Inspection Station, Kanagawa Prefectural Government
KSLHS: Kanagawa Shonan Livestock Hygiene Service Center
PGS: Paternal grand sire
MGS: Maternal grand sire
OR: Odds ratio
BoLA: Bovine leukocyte antigen
